# A Novel, Simple, and Reliable Spectrophotometric Determination of Total Hexavalent Chromium by Complexation with a New Reagent of Thiazole Linked to 2H-Chromen-2-One

**DOI:** 10.1155/2023/2042221

**Published:** 2023-02-27

**Authors:** Abdullah Akhdhar

**Affiliations:** Department of Chemistry, College of Science, University of Jeddah, Jeddah, Saudi Arabia

## Abstract

Hexavalent chromium is a known environmental contaminant and carcinogen. In the current work, a simple, rapid, and reliable direct spectrophotometric method was used for the determination of total Cr (VI) in environmental samples. Acid-base equilibria and ionization constant (pK_a_) of the new reagent 3-(2-(2-(4-(trifluoromethyl)benzylidene)hydrazineyl) thiazol-4-yl)-2H-chromen-2-one (thiazole linked to 2H-chromen-2-one, TFZ) were investigated. The value of pK_a_ for the reagent was found to be 7.6 which was initially reported. The reaction of the TFZ ligand with Cr (VI) was optimized to produce a highly absorbent complex at 370 nm and pH 7.0 within 1 min. With a correlation coefficient of 0.9994, the linear concentration range ranges from 2 to 20,000 ng·mL^−1^. The detection limit and quantification limit were 0.73 and 2.43 ng·mL^−1^, respectively. The method has high precision with relative standard deviations less than 1.0 and high accuracy with recovery of 100 ± 2%. A large excess of cations and anions did not interfere with the determination of Cr (VI). The proposed method was successfully applied to the determination of Cr (VI) in cement samples. The current method could be useful for the routine analysis of Cr (VI) in environmental labs.

## 1. Introduction

Contamination of the environment with heavy metals is a growing concern due to their toxicity to humans, animals, including fish, and the ecosystem. In 1797, the French scientist Louis Vauquelin identified an element whose colour changed as it existed in different chemical forms. Because one of the compounds that included this element was green and “chroma,” meaning “colour,” the element was named chromium (Cr) [1]. Chromium has been identified as a poisonous and carcinogenic metal. Chromium is employed in steel manufacturing and polishing, alloying, electroplating, leather tanning and finishing, dyes and mordants, catalysts, oxidants, adhesives, paints, and wood preservatives [2]. In the environment, chromium can be found in two stable forms: Cr (VI) and Cr (III). Cr (III) is less toxic and less soluble than Cr (VI), whereas Cr (VI) is exceedingly toxic and extremely soluble [3]. Cr (VI) is roughly 100–300 times more hazardous than Cr (III) which has a high penetration capacity for biofilm, which can be transformed into a variety of reactive intermediates, resulting in DNA mutation and cancer [4, 5]. It also causes liver damage, lung congestion, inflammation, skin irritation, and ulcers [5]. According to the World Health Organization (WHO) and the Environmental Protection Agency (EPA), the maximum permissible level of chromium in drinking water is 50 *μ*g·L^−1^ and 100 *μ*g·L^−1^, respectively [6, 7]. Directive 2003/53/EC of the European Union (EU) mandated the control and limitation of chromium levels in cement containing more than 2 mg·kg^−1^ of Cr (VI).  Table 1 shows the recommended maximum permissible limit of chromium in fish, food, and animals' meat.

Several analytical techniques, such as electrothermal atomic absorption spectroscopy, are used to determine chromium [8], flame atomic absorption spectrometry (FAAS) [9], UV-Vis spectrophotometry [10], atomic absorption spectroscopy (AAS), and inductively coupled plasma optical emission spectroscopy/inductively coupled plasma mass spectrometry “(ICP-OES/ICP-MS) [2, 11]. AAS and ICP-OES/ICP-MS are both capable of detecting only total chromium without separating between different chromium species. As a result, various chromium species were separated using ion chromatography (IC) or high-performance liquid chromatography (HPLC) prior to detection using ICP-OES/ICP-MS and AAS [12, 13]. However, absorption spectrophotometry is more suitable and reliable for routine chemical analysis due to its widespread availability and relatively inexpensive cost of equipment, as well as the stability of its processes and the precision of its methodologies [14]. Moreover, spectrophotometry has opportunities not just for enhancing selectivity but also for improving sensitivity.

**Figure 1:**
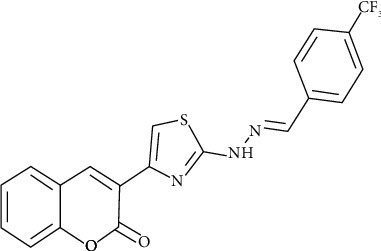
Chemical structure of 3-(2-(2-(4-(trifluoromethyl)benzylidene)hydrazineyl)thiazol-4-yl)-2H-chromen-2-one(3a) (thiazole linked to 2H-chromen-2-one, TFZ) [18].

Thus, the aim of current work is to develop a new spectrophotometric method with a highly sensitive and wide linear range for Cr (VI) determination by complexation with the novel ligand 3-(2-(2-(4-(trifluoromethyl)benzylidene)hydrazineyl)thiazol-4-yl)-2H-chromen-2-one(3a) (thiazole linked to 2H-chromen-2-one, TFZ, Figure 1). Several experimental parameters which influence the formation of the Cr (VI)-THZ complex were investigated and optimized. Finally, the proposed method was applied to determine total soluble Cr (VI) in various cement, water, and soil samples.

## 2. Experimental Setup

### 2.1. Reagents and Chemicals

Potassium chromate was purchased as a yellow powder from Sigma Aldrich (USA). Iron (II) sulphate 7-hydrate, manganese sulphate monohydrate, copper sulphate, and cadmium nitrate hydrated were bought as powders from TECHNO PHARMACHEM (India) and nickel sulphate extra pure from Loba. Chemi (India) were used as powders for foreign ions. Sodium phosphate dibasic dihydrate (Sigma Aldrich, USA) and sodium dihydrogen orthophosphate dihydrate (CDH, India) were used to prepare the solution in different pH levels. Ethanol was obtained from Sasol, KSA, which was used for the preparation of the reagent. Glassware and polyethylene containers were soaked in 10% nitric acid and washed with deionized water to eliminate the risk of contamination. Nitric acid was supplied by Sigma Aldrich (USA), while hydrochloric acid was obtained from Honeywell (USA) and hydrogen peroxide solution was purchased from Fluka (Germany). All other chemicals were purchased from Merck (Darmstadt, Germany) and used without further purification. For the manufacture of aqueous solutions, double-distilled water was employed.

### 2.2. Instrumentation

The absorbance spectra were determined using a UV-visible spectrophotometer from PG Instruments, U.K., with a matched pair of 1 cm quartz cuvettes at a detecting wavelength between 250 and 700 nm. The buffer solutions' pH was measured with a pH meter from Eutech. Utilizing standard buffer solutions of pH 4.0, 7.0, and 10.0 at room temperature, the pH meter was calibrated.

### 2.3. Synthesis and Preparation of TFZ Ligand Solution

According to the publication of our research group, the TFZ ligand was produced [18].

### 2.4. Method for pK_a_ Determination

The pK_a_ of the TFZ ligand was determined using a method created by Pandey et al. [19]. A preliminary investigation was conducted to estimate the pK_a_ using the inflection approach. A graph of absorbance against pH was created. This curve inflection point was cited as a rough estimate of the pK_a_ value. The second approach consisted of determining the wavelengths of maximum absorption from the spectra of species with extreme pH values (pH = 2 and pH = 8 in this situation). The plot of the absorbance vs. pH at these wavelengths was applied. The pKa was determined by measuring the pH of the place where the two linear curves intersected at these wavelengths.

### 2.5. Preparation of Standard and Working Solutions of Cr (VI)

The Cr (VI) solution was prepared by dissolving 100 mg of potassium chromate sample in 100 mL distilled water. The working solutions of the Cr (VI) were prepared by dilution in the same solvent.

### 2.6. Procedure of Complex Formation

The formed coloured complex solution was simply and precisely prepared. A 0.1 mL of Cr (VI) was mixed with 9.8 mL of phosphate buffer (pH 7), and then 0.1 mL of ligand was added. The complex was rapidly formed after 1 min of mixing solutions.

### 2.7. Environmental Samples

Several samples were collected; three ground water samples from different places of Saudi Arabia were collected. Soil (surface) samples were collected from five different locations in Jeddah city, Saudi Arabia. Two cement samples were purchased from the local market.

### 2.8. Digestion of Soil and Cement Samples

The digestion of the soil and cement samples was performed according to the previously reported method with little modification [20, 21]. Typically, 0.5 g of samples were dissolved in a mixture of 15 mL nitric acid and 5 mL hydrochloric acid followed by the addition of 2 mL hydrogen peroxide and 2 mL water. The sample was placed in a suitable beaker and covered by a clock glass. The beaker was heated in an oil bath. The specified temperature profile included reaching 180 ± 5°C in less than 5.5 minutes and remaining at that temperature for 9.5 minutes to accomplish the desired reactions. After cooling, the beaker content was filtered and was ready for starting analysis.

## 3. Results and Discussion

### 3.1. Investigation of Acid-Base Equilibria of the TFZ Ligand

To accomplish this, the absorbance of the TFZ ligand was evaluated in buffers ranging in pH from 2.0 to 12.0. The buffer solutions were prepared by using 0.1 mol·L^−1^ sodium phosphate dibasic dihydrate (NaH_2_PO_4_) and sodium dihydrogen orthophosphate (Na_2_HPO_4_). Primary stock solution (1000 *µ*g·mL^−1^) of the TFZ ligand was prepared in ethanol. All samples were analyzed after 10 *µ*g·mL^−1^ working solutions were produced in corresponding buffers from primary stock solutions.

**Figure 2:**
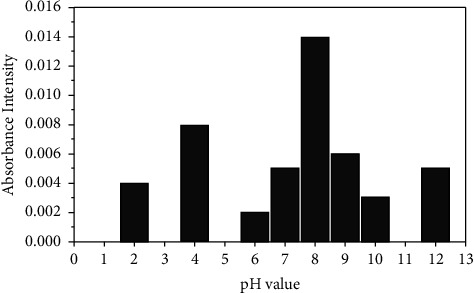
Absorbance of the TFZ ligand at 370 nm in respective buffer solutions of pH 2.0–12.0.

To calculate the ionization constant (pK_a_) of TFZ ligand spectra, two graphical methods were applied. The simple first one was used to calculate a rough value of pK_a_.  Figure 2 shows the absorbance diagram of the TFZ ligand in buffer solutions of various pH values at a maximum wavelength of 400 nm. It is clear from the figure that the TFZ ligand exhibits pH dependent UV-absorption, and the high absorption was achieved at pH 8. Therefore, the pK_a_ value of 8 could be considered an approximate ionization constant for the TFZ ligand.

Another method was used to calculate the precise pK_a_ value of TFZ (L). The approach involved measuring the spectra of species with extreme pH levels (pH = 2 and pH = 8 in this instance) to identify the wavelengths of maximum absorption. As observed, the absorbance spectrum of the acidic solution exhibited a peak at 515 nm (HL^+^), while the peak of the more basic solution occurred at 400 nm (L). The pKa was obtained by determining the pH of the point of intersection, and it was found to be 7.6.

### 3.2. Cr (VI) Complexation with the TFZ Ligand

#### 3.2.1. Complexation Equilibria of Cr (VI) with TFZ

**Figure 3:**
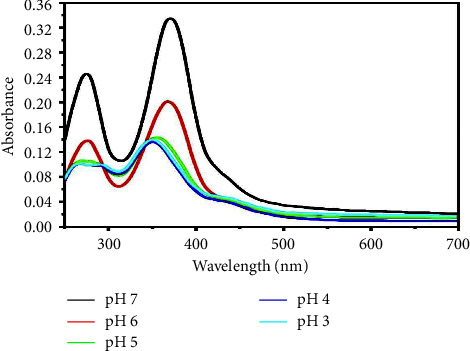
Effect of pH on the Cr (VI)-TFZ complex.

**Figure 4:**
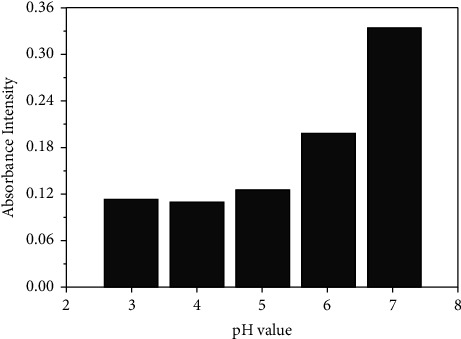
Effect of pH on the Cr (VI)-TFZ complex at 370 nm.

The pH study is essential since it can have a significant effect on the formation of metal-ligand complexes as well as the result of complex extraction. The effect of pH on the formation of the Cr (VI)-TFZ complex in the mixed water-ethanol medium was simply investigated by mixing equal volumes (0.1 mL) of each ligand and metal of 1000 *µ*g·mL^−1^ and 9.8 mL of buffers with different pHs from acidic to basic media. After a waiting period of 1 min to complete the complex reaction, the absorbance was measured. It was found that the best pH for perfume complex formation and to get a maximum light response was at pH = 7 as shown in Figures 3 and 4. In the strong acidic medium, the complex's absorbance is low, and this may be due to ligand (Schiff base) instability.

**Figure 5:**
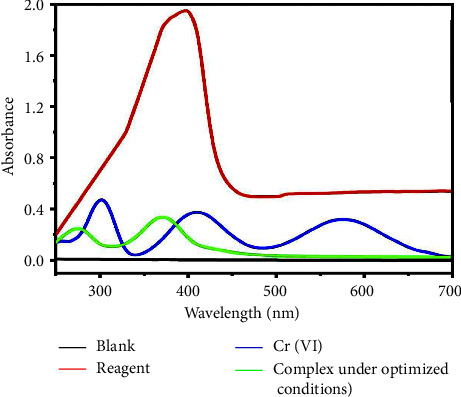
A comparison between blank, TFZ, Cr (VI), and the complex under optimized conditions.

A comparison between phosphate buffer blank solution, TFZ ligand solution, Cr (VI) solution, and TFZ-Cr (VI) complex solution under the optimized conditions of pH 7 is shown in Figure 5. It is clear that the maximum absorbance of the ligand (at 400 nm) and Cr (VI) (at 435 nm) was shifted to the maximum absorbance at 370 nm, proving the complex formation between Cr (VI) and TFZ. One can assume that the neutral form of the TFZ (L) is the prevalent ligand species in the pH of complexation with the liberation of one proton.

#### 3.2.2. Effect of Sequence on Complex Formation

As shown in Table 2, the best sequence of solution addition (in complex formation) to achieve maximum absorbance is Cr (VI) (M) + phosphate buffer (B) + TFZ (L). This result is expected because buffered ligand solution is a suitable medium to form complexes with better colour intensity.

#### 3.2.3. Effect of Time on Complex Formation

Five different times between 1 and 15 minutes were studied. The reaction was very fast, and the highest absorbance of the complex was obtained after 1 min of mixing metal with the ligand.

#### 3.2.4. The Mole Ratio of the Complex

**Figure 6:**
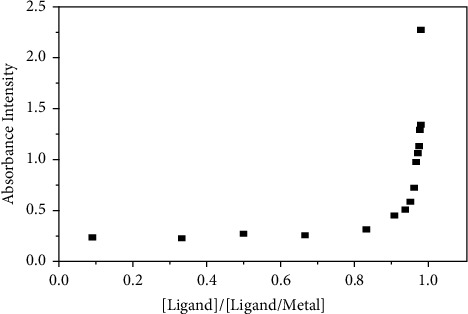
Determination of complex compositions by the mole ratio method.

This method was performed by mixing different volumes of TFZ ligand solution with the same volume of Cr (VI) ions solution each time; the absorbance of resulted complex solutions was recorded after passing reaction time of 1 min. It is important to prepare each of metal ions and ligand solutions in equal concentrations. As shown in Figure 6, the M : L mole ratio in complex equals to 1 : 1.

#### 3.2.5. Calibration Characteristics

By mixing different concentrations of Cr (VI) with constant concentration of the ligand and leaving the solution mixture for 1 minute to make the reaction complete, the following analytical results were recorded, and figures of merit for the determination of Cr (VI) are shown in Table 3. By assessing a series of five solutions with a 10 ng·mL^−1^ Cr (VI) concentration, the reproducibility and accuracy of the procedure were determined. The relative standard deviation (RSD) was observed to be 0.975, and high accuracy with recovery values of 100 ± 2% was observed.

#### 3.2.6. Study of Interferences

To determine whether or not the proposed method is effective, the effects of a number of ions that are frequently found in environmental samples were investigated. The tolerance of the method to foreign ions was investigated with solutions containing 1000 *µ*g·mL^−1^ of expected interfering cations and anions in the presence of 10 ng·mL^−1^ Cr (VI). The tolerance level for a given ion was the variation of absorbance readings from the predicted value by more than ± 2%. Experimental results showed that Cd, Cu, Cr (III), Fe, Mn, and Ni have no influence on the determination of Cr (VI).

#### 3.2.7. Applications

The promise of TFZ as a reagent for direct spectrophotometric detection of total hexavalent chromium prompted us to examine the applicability of the method to the study of soluble hexavalent chromium in environmental samples at 370 nm. The determination of total hexavalent chromium was conducted in different environmental samples: three ground water samples, five soil (surface) samples, and two cement samples. The results of the analysis of water and soil samples showed that they were below the LOD value. The obtained values for Cr (VI) concentrations in cement samples using the proposed analytical method were found to be 4%. In the precision study, five analyses were performed for each sample. A good precision of the proposed method was obtained, which allow the application of the method to the routine analysis of cement.

To confirm the analytical characteristics of the current method compared to the previous published methods for Cr (VI) analysis in cement, all data which are very rare are collected in Table 4. The sensitivity of the current method is comparable to others.

## 4. Conclusion

The current analytical method for the determination of Cr (VI) content in environmental samples has proved to be reliable, simple, and rapid. The method has the ability to be performed as a quick test for determining hexavalent chromium in cement. Numerous further advantages of the current method include the fact that the complexion reagent can be made on any scale with great purity and a specific chelation with Cr (VI) in neutral medium, there is no need for a pretreatment step to enhance the analyte concentration, and it is applicable to a wide range of analyte concentrations. Therefore, the current method could be used for selective trace determination of chromium in several matrices.

## Figures and Tables

**Table 1:** The recommended maximum permissible limit of chromium in fish, food, and animals' meat.

Food matrix	Maximum permissible limit (MPL) (mg/kg)	Reference
Fish	0.65–4.35	[8]
Food	0.01–13	[9]
Animals' meat	0.05	[10]

**Table 2:** Effect of sequence addition on complex colour intensity.

Sequence of addition	Absorbance
Metal (M) + buffer (B) + ligand (L)	0.335
Ligand (L) + buffer (B) + metal (M)	0.291

**Table 3:** Figures of merit for the determination of Cr (VI)-TFZ complexation.

Parameter	Value
Absorption wavelength for complexation (nm)	370
Detection limit (ng mL^−1^)	0.73
Quantification limit (ng mL^−1^)	2.43
Correlation coefficient (R)	0.9994
Linear working range (ng mL^−1^)	2–20000

**Table 4:** Methods for measuring Cr (VI) in cement using a variety of chromogenic agents: a comparison.

Method	Matrix	Reagent	LOD (mg/kg)	Reference
Spectrophotometric	Cement	Diphenylcarbazide	0.0010	[22]
Spectrophotometric	Cement	Variamine blue	0.0500	[22]
Spectrophotometric	Cement	1, 5-diphenylcarbazide	0.0013	[22]
Spectrophotometric	Cement	Thiazole linked to 2H-chromen-2-one	0.0007	This study

## Data Availability

The data supporting the current study are available from the corresponding author upon request.

## References

[1] Long H., Huang X., Liao Y., Ding J. (2021). Recovery of Cr (VI) from tannery sludge and chrome-tanned leather shavings by Na 2 CO 3 segmented calcination. *Journal of Environmental Chemical Engineering*.

[2] Zhao K., Ge L., Wong T. I., Zhou X., Lisak G. (2021). Gold-silver nanoparticles modified electrochemical sensor array for simultaneous determination of chromium(III) and chromium(VI) in wastewater samples. *Chemosphere*.

[3] Coetzee J. J., Bansal N., Chirwa E. M. N. (2020). Chromium in environment, its toxic effect from chromite-mining and ferrochrome industries, and its possible bioremediation. *Exposure and Health*.

[4] Lv Z., Tan X., Wang C., Alsaedi A., Hayat T., Chen C. (2020). Metal-organic frameworks-derived 3D yolk shell-like structure Ni@carbon as a recyclable catalyst for Cr(VI) reduction. *Chemical Engineering Journal*.

[5] Miretzky P., Cirelli A. F. (2010). Cr (VI) and Cr (III) removal from aqueous solution by raw and modified lignocellulosic materials: a review. *Journal of Hazardous Materials*.

[6] Tuzen M., Elik A., Altunay N. (2021). Ultrasound-assisted supramolecular solvent dispersive liquid-liquid microextraction for preconcentration and determination of Cr (VI) in waters and total chromium in beverages and vegetables. *Journal of Molecular Liquids*.

[7] Martín-domínguez A., Rivera-huerta M. L., Pérez-castrejón S. (2018). Chromium removal from drinking water by redox-assisted coagulation: chemical versus electrocoagulation. *Separation and Purification Technology*.

[8] Rakib M. R. J., Jolly Y. N., Enyoh C. E. (2021). Levels and health risk assessment of heavy metals in dried fish consumed in Bangladesh. *Scientific Reports*.

[9] Fluoride H. (2003). Potential for human exposure. *Exposure*.

[10] Amfo-otu R. (2016). Meat contamination through singeing with scrap tyres in akropongakuapem. https://www.researchgate.net/publication/280240333_MEAT_CONTAMINATION_THROUGH_SINGEING_WITH_SCRAP_TYRES_IN_AKROPONGAKUAPEM_ABATTOIR_GHANA.

[11] Dokpikul N., Chaiyasith W. C., Sananmuang R., Ampiah-bonney R. J. (2018). Surfactant-assisted emulsi fi cation dispersive liquid-liquid microextraction using 2-thenoyltri fl uoroacetone as a chelating agent coupled with electrothermal atomic absorption spectrometry for the speciation of chromium in water and rice samples. *Food Chemistry*.

[12] Khoshmaram L., Mohammadi M. (2021). Combination of a smart phone based low-cost portable colorimeter with air-assisted liquid-liquid microextraction for speciation and determination of chromium (III) and (VI). *Microchemical Journal*.

[13] Ahmad W., Bashammakh A. S., Al-Sibaai A., Alwael H., El-Shahawi M. (2016). Trace determination of Cr (III) and Cr (VI) species in water samples via dispersive liquid-liquid microextraction and microvolume UV – vis spectrometry . Thermodynamics , speciation study. *Journal of Molecular Liquids*.

[14] Biswas P., Karn A. K., Balasubramanian P., Kale P. G. (2017). Biosensor for detection of dissolved chromium in potable water: a review. *Biosensors and Bioelectronics*.

[15] Meng Q., Fan Z. T., Buckley B. (2011). Development and evaluation of a method for hexavalent chromium in ambient air using IC-ICP-MS. *Atmospheric Environment*.

[16] Markiewicz B., Komorowicz I., Sajnóg A., Belter M., Barałkiewicz D. (2015). Chromium and its speciation in water samples by HPLC/ICP-MS – technique establishing metrological traceability: a review since 2000. *Talanta*.

[17] Idriss K. A., Sedaira H., Dardeery S. (2013). Spectrophotometric determination of water-soluble hexavalent chromium and determination of total hexavalent chromium content of portland cement in the presence of iron (III) and titanium (IV) using derivative ratio spectrophotometry. *American Journal of Analytical Chemistry*.

[18] Saleh T. S., Aqlan F. M., Almaghrabi O. A., Al-Bogami A. S. (2022). Mechanochemical rapid synthesis of novel thiazoles linked to 2H-Chromen-2-one moiety. *Heterocycles*.

[19] Pandey M. M., Jaipal A., Kumar A., Malik R., Charde S. Y. (2013). Determination of pKa of felodipine using UV-Visible spectroscopy. *Spectrochimica Acta Part A: Molecular and Biomolecular Spectroscopy*.

[20] Pezzin S. H., Rivera J. F. L., Collins C. H., Collins K. E. (2004). Reduction of trace quantities of chromium(VI) by strong. *Acids*.

[21] Shaibur M. R., Shamim A. H., Huq S. M. I., Kawai S. (2010). Comparison of digesting capacity of nitric acid and nitric acid-perchloric acid mixture and the effect of lanthanum chloride on potassium measurement. *Nature and Science*.

[22] Ramírez-Quesada M. M., Venegas-Padilla J., Sibaja-Brenes J. P., Calderón-Jiménez B. (2021). New advances in the method validation, extraction methods and measurement uncertainty for the determination of water-soluble hexavalent chromium in hydraulic cement. *Talanta*.

